# lncRNA-MIAT rs9625066 polymorphism could be a potential biomarker for ischemic stroke

**DOI:** 10.1186/s12920-024-01830-w

**Published:** 2024-02-21

**Authors:** Yin-Hua Weng, Jie Chen, Wen-Tao Yu, Yan-Ping Luo, Chao Liu, Jun Yang, Hong-Bo Liu

**Affiliations:** 1 Department of Laboratory Medicine, The Second Affiliated Hospital of Guilin Medical University, Guilin, China; 2 Department of Laboratory Medicine, Affiliated Hospital of Guilin Medical University, Guilin, China; 3 School of Clinical Medicine, Guilin Medical University, Guilin, China; 4 College of Medical Laboratory Science, Guilin Medical University, Guilin, China; 5 Guangxi Key Laboratory of Metabolic Reprogramming and Intelligent Medical Engineering for Chronic Diseases, Guangxi Clinical Research Center for Diabetes and Metabolic Diseases, The Second Affiliated Hospital of Guilin Medical University, Guilin, China

**Keywords:** Ischemic stroke, MIAT, Polymorphism, lncRNA, rs9625066

## Abstract

**Background:**

Ischemic stroke (IS) is a common and serious neurological condition that is highly fatal but so far no early diagnostic markers are available. Myocardial infarction-associated transcript (MIAT) is a long non-coding RNA (lncRNA) that could lead to IS by inducing autophagy and apoptosis in neuronal cells. However, there has been no report on the link between susceptibility to IS and the single-nucleotide polymorphisms (SNPs) of MIAT. This study aimed to investigate the association between MIAT gene polymorphisms and IS risk.

**Methods:**

A total of 320 IS patients and 310 age-, sex- and race-matched controls were included in this study. Four polymorphisms (rs2157598, rs5761664, rs1894720, and rs9625066) were genotyped by using SNPscan technique.

**Results:**

Among the 4 polymorphisms of MIAT, only rs9625066 was associated with IS risk (CA vs. CC: adjusted OR = 0.55, 95% CI, 0.37–0.85, *P* = 0.006; AA vs. CC: adjusted OR = 0.39, 95% CI, 0.16–0.94, *P* = 0.036; (AA + CA vs. CC: adjusted OR = 0.53, 95% CI, 0.35–0.80, *P* = 0.002; A vs. C adjusted OR = 0.59, 95% CI, 0.42–0.82, *P* = 0.002). Haplotype analysis showed a 1.32-fold increase (95% CI, 1.05–1.67, *P* = 0.017) in IS risk for rs2157598-rs5761664-rs1894720-rs9625066 (A-C-G-C). Logistic regression analysis identified some independent impact factors for IS including rs9625066 AA/AC, TC, TG, HDL-C (*P* < 0.05).

**Conclusion:**

The rs9625066 polymorphism of MIAT might be associated with IS susceptibility in Chinese population, in which AA/CA plays a protective role in IS, whereas the CC genotype increases the risk of developing IS, suggesting it might be a marker predictive of IS risk.

## Introduction

Ischemic stroke (IS), also known clinically as cerebral infarction, represents a common and serious condition in which brain blood flow is reduced or interrupted due to stenosis or occlusion of arteries, resulting in ischemic necrosis of brain tissue in the affected area [1, 2]. IS is clinically characterized by sudden onset, rapid progression, and high mortality and disability rates, among others [3]. IS reportedly accounted for up to 80% of cardiovascular and cerebrovascular diseases and has been a subject of active studies. The risk factors associated with IS include age, gender, dietary habits, hyperglycemia, hyperlipidemia, and hypertension, *etc.* [4]. In recent years, the pathogenesis of IS has been found to be intimately linked to genetic factors, such as transcription, expression and variation of some genes [5, 6].

Long non-coding RNAs (lncRNAs), as an important class of non-coding RNAs, have been extensively studied in a wide array of biological processes and mounting evidence showed that they play a key role in the development of a multitude of diseases [7]. The lncRNA MIAT (Myocardial Infarction Associated Transcript) is located on chromosome 22q12.1 and was originally found to be a lncRNA associated with myocardial infarction [8]. However, recent studies revealed that the lncRNA MIAT was not only associated with cardiovascular diseases, but also bore close correlation with neurological conditions, including IS [9–11]. Guo et al. found that MIAT expression was increased in IS rats and in PC12 cells injured by oxygen-glucose deprivation/reoxygenation (OGD/R) and the MIAT over-expression exacerbated IS by promoting autophagy and apoptosis in neuronal cells through upregulating REDD1 (DNA damage responses 1) expression [12]. Li et al. demonstrated that MIAT overexpression increased infarct volume and induced neuronal apoptosis in the rat middle cerebral artery occlusion (MCAO) model [13]. These findings support that the MIAT might be implicated in the pathological process of IS.

At present, lncRNA-associated single-nucleotide polymorphisms (SNPs) have been shown to potentially influence the susceptibility to IS. For example, the rs2240183 CT/CC genotype and the C allele in the promoter of the lncRNA TUG1 gene were found to be associated with an increased risk of IS [14], whereas the rs1194338 AC/AA genotype in the promoter of the lncRNA MALAT1 might be a protective factor against IS [15]. In this study, we, by employing a case-control method, investigated the correlation between SNPs in the MIAT gene (rs2157598, rs5761664, rs1894720, and rs9625066) and the susceptibility to IS in Chinese population.

## Materials and methods

### Study population

Three hundred twenty samples in the case group came from patients who presented at the Affiliated Hospital of Guilin Medical College in Guangxi, China, from June 2020 to June 2021. Their clinical symptoms, CT and MRI findings satisfied the diagnostic criteria of IS proposed by the 2014 Chinese Diagnostic Guidelines for Acute Ischemic Stroke, and the candidate subjects were excluded if their strokes were caused by traumas, brain tumors, cerebral parasitosis, or other diseases. Three hundred ten cases in the control group were sex- and age-matched healthy subjects recruited at the same period of time, and those with cardiovascular and cerebrovascular diseases, tumors, neurological diseases, and hereditary diseases were excluded. The following data were collected: sex, age, blood glucose level, triglycerides (TG), total cholesterol (TC), high-density lipoprotein cholesterol (HDL-C), and low-density lipoprotein cholesterol (LDL-C).

### Ethics

This study was conducted in accordance with the guidelines of the Helsinki Declaration and approved by the Ethics Review Committee of Affiliated Hospital of Guilin Medical University, and all subjects who participated in the study signed an informed consent form.

### Screening of SNPs and the basis of selection

We searched for MIAT gene-related information in the NCBI database. Four SNPs (rs2157598, rs5761664, rs1894720 and rs9625066) were selected against the following criteria: (1) loci with minor allele frequency (MAF) > 5% in the Chinese Han population, (2) SNPs with potential functions in the promoter region of the MIAT gene as predicted by computerized analyses, and (3) loci that had been reported in the literature and have been studied in other diseases. Our selection criteria were to ensure that data were more in line with the genetic profile of the target population, with focus directed at SNPs that bore correlations with biological functions and potential disease, thus increasing the power of the study and the interpretability of the results.

### DNA extraction and genotyping

3–5 ml of peripheral blood samples were harvested and genomic DNA was extracted using a DNA extraction kit (DP318, Qiagen, China). Genotyping was performed by using the SNPscan™ Multiple SNP typing kit on the ABI3730XL amplifier (PE Applied Biosystems, Foster City, CA, USA), and the data collected were analyzed using GeneMapper 4.1 (Applied Biosystems, USA). Testing was repeated on 10% of randomly selected DNA samples to verify the reproducibility of the experiments, and a consistency rate of > 99% was required.

### Statistical analysis

All statistical analyses were performed by employing IBM SPSS 20.0 software package (SPSS Inc., Chicago, IL, USA). Continuous variables were expressed as mean ± standard deviation (SD) and analyzed using t-test. Hardy-Weinberg equilibrium (HWE) and categorical data, such as gender and diabetes, were analyzed using chi-square test. Logistic regression was utilized to analyze the association between SNP and IS. Risk was assessed by calculating odds ratio (OR) and 95% confidence intervals (95% CI). OR was adjusted for sex, age, presence of diabetes, TC, TG, HDL-C, and LDL-C. SHEsis software was employed for linkage disequilibrium (LD) and haplotype analysis. Difference was considered to be statistically significant when a *P* < 0.05.

## Results

### Characteristics of the study population

The features of the study population are shown in Table 1. There were no significant differences in the distribution of cases and controls in terms of age, sex and presence of diabetes mellitus (*P* > 0.05). Levels of TC, TG and LDL and lower levels of HDL were higher in IS patients than in controls (all *P* < 0.05).

**Table 1:** Clinical characteristics of the study population

Variables	Controls, *n* = 310	IS patients, *n* = 320	*P* value
Gender (M/F)	165/145	187/133	0.188
Age, years (Mean ± SD)	62.42 ± 7.02	63.42 ± 8.52	0.105
Diabetes mellitus (%)	50 (19.2)	66 (20.6)	0.175
TC (mmol/L)	4.02 ± 1.01	4.61 ± 1.21	< 0.001
TG (mmol/L)	1.62 ± 0.95	2.01 ± 1.61	< 0.001
HDL-C (mmol/L)	1.60 ± 0.93	1.04 ± 0.26	< 0.001
LDL-C (mmol/L)	2.54 ± 0.86	2.97 ± 1.18	< 0.001

*IS* ischemic stroke, *SD* Standard deviation, *M* Male, *F* Female, *TC* Total cholesterol, *TG* Triglyceride, *HDL-C* High density lipoprotein-cholesterol, *LDL-C* Low density lipoprotein-cholesterol

### Association between MIAT polymorphisms and IS risk

In this study, all four SNPs were found to have three genotypes as analyzed by SNPscan technique (Table 2). The distribution of genotypes in the case and control groups followed the HWE (*P* > 0.05). The A allele of rs9625066 was found to be associated with a lower risk of IS as compared with the C allele (A vs. C adjusted OR = 0.59, 95% CI, 0.42-0.82, *P* = 0.002), and that AA genotypes, CA genotypes, and the dominant model bore a correlation with the lower risk for IS (CA vs. CC: adjusted OR = 0.55, 95% CI, 0.37-0.85, *P* = 0.006; AA vs. CC: adjusted OR = 0. 39, 95% CI, 0.16–0.94, *P* = 0.036; AA + CA vs. CC: adjusted OR = 0.53, 95% CI, 0.35–0.80, *P* = 0.002). No significant associations were observed between the rs2157598, rs5761664, and rs1894720 polymorphisms and the risk of IS (*P* > 0.05). Subsequent correction for multiple comparisons revealed that the initially observed protective effect of the AA genotype on IS has lost statistical significance. Therefore, further studies with a larger sample size are needed to confirm these associations.

**Table 2:** Association between MIAT polymorphisms and IS risk

Polymorphisms	Controls, *n* = 310	HEW, *P*^a^	IS patients, *n* = 320	HEW, *P*^b^	OR (95%CI)^†^	*P*^†^	*P*_(BH)_
rs2157598		0.831		0.642			
AA	99 (31.9)		112 (35.0)		1.00(ref)		
AT	154 (49.7)		158 (49.4)		0.82 (0.53-1.26)	0.370	0.663
TT	57 (18.4)		50 (15.6)		1.07 (0.60-1.93)	0.811	0.901
A	352 (56.8)		382 (59.7)		1.00(ref)		
T	268 (43.2)		258 (40.3)		0.99 (0.75-1.31)	0.954	0.954
Dominant model							
AA	99 (31.9)		112 (35.0)		1.00(ref)		
TT + TA	211 (68.1)		208 (65.0)		0.88 (0.58-1.32)	0.530	0.663
Recessive model							
AA + TA	253 (81.6)		270 (84.4)		1.00(ref)		
TT	57 (18.4)		50 (15.6)		1.21 (0.71-2.04)	0.484	0.663
rs5761664		0.798		0.824			
CC	132 (42.6)		146 (45.6)		1.00(ref)		
CT	139 (44.8)		139 (43.4)		0.86 (0.57-1.31)	0.488	0.663
TT	39 (12.6)		35 (10.9)		1.55 (0.80-2.99)	0.192	0.433
C	403 (65.0)		431 (67.3)		1.00(ref)		
T	217 (35.0)		209 (32.7)		1.1 (0.82-1.47)	0.515	0.663
Dominant model							
CC	132 (42.6)		146 (45.6)		1.00(ref)		
TT + TC	178 (57.4)		174 (54.4)		0.97 (0.66-1.43)	0.876	0.922
Recessive model							
CC + TC	271 (87.4)		285 (89.1)		1.00(ref)		
TT	39 (12.6)		35 (10.9)		1.67 (0.90-3.12)	0.106	0.303
rs1894720		0.711		0.143			
GG	118 (38.1)		133 (41.6)		1.00(ref)		
GT	144 (46.5)		137 (42.8)		0.78 (0.51-1.19)	0.250	0.500
TT	48 (15.5)		50 (15.6)		1.48 (0.82-2.68)	0.195	0.433
G	380 (61.3)		403 (63.0)		1.00(ref)		
T	240 (38.7)		237 (37.0)		1.11 (0.83-1.47)	0.490	0.663
Dominant model							
GG	118 (38.1)		133 (41.6)		1.00(ref)		
TT + GT	192 (61.9)		187 (58.4)		0.91 (0.61-1.36)	0.656	0.772
Recessive model							
GG + GT	262 (84.5)		270 (84.4)		1.00(ref)		
TT	48 (15.5)		50 (15.6)		1.70 (0.98-2.93)	0.058	0.232
rs9625066		0.732		0.354			
CC	174 (56.1)		202 (63.1)		1.00 (ref)		
CA	118 (38.1)		101 (31.6)		0.55 (0.37-0.85)	0.006	0.040
AA	18 (5.80)		17 (5.30)		0.39 (0.16-0.94)	0.036	0.180
C	466 (75.2)		505 (78.9)		1.00 (ref)		
A	154 (24.8)		135 (21.1)		0.59 (0.42-0.82)	0.002	0.020
Dominant model							
CC	174 (56.1)		202 (63.1)		1.00 (ref)		
AA + CA	136 (43.9)		118 (36.9)		0.53 (0.35-0.80)	0.002	0.020
Recessive model							
CC + CA	292 (94.2)		303 (94.7)		1.00 (ref)		
AA	18 (5.80)		17 (5.30)		0.49 (0.21-1.16)	0.106	0.303

*OR* odds ratio, represents a statistical measure comparing the probability of events occurring in two groups, *HWE* Hardy-Weinberg equilibrium, refers to the principle of stable genotype frequencies in a genetic population

^a^Controls group

^b^IS patients group

^†^Adjusted by age, gender, diabetes mellitus, TG, TC, LDL-C and HDL-C; *P*_(BH):_*P* values corrected by Benjamin-Hochberg (B-H) method

### Haplotype analysis of MIAT polymorphisms and IS risk

**Fig. 1:**
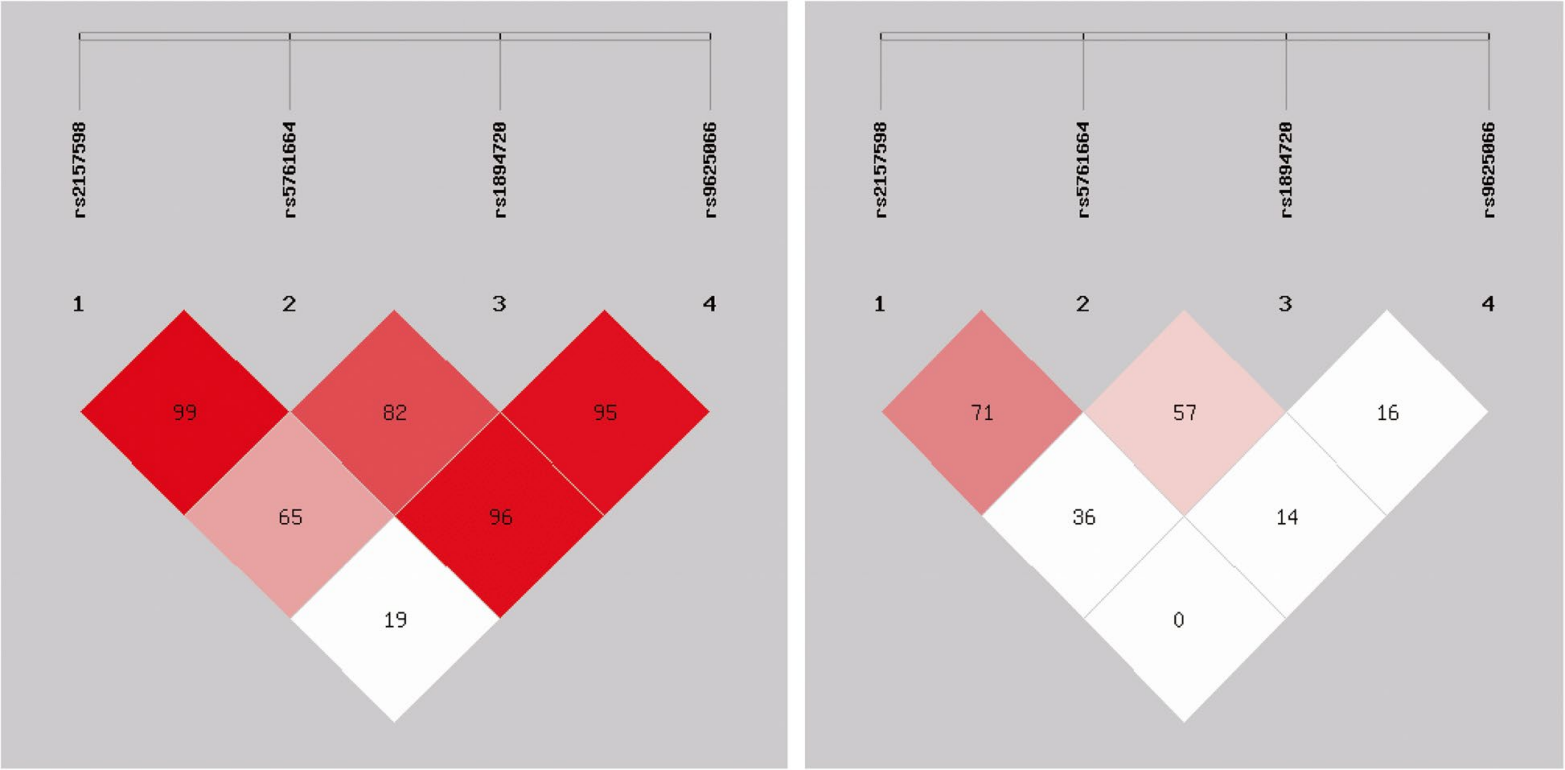
Linkage disequilibrium (LD) indicated there was a correlation between specific genotypes. LD test for four polymorphisms. rs2157598 and rs5761664 were found to have strong LD (D' = 0.99, *r*^2^ = 0.71)

We assessed the haplotype frequencies of the four SNPs in the MIAT gene in IS patients and controls by using the online SHEsis software (Table 3). The results revealed that rs2157598-rs5761664-rs1894720-rs9625066 (A-C-G-C) were the predominant haplotypes in IS patients and healthy controls. We also found that the elevated risk of IS was associated with the presence of haplotypes (A-C-G-C) (OR = 1.32,95% Cl, 1.05–1.67, *P* = 0.017). The presence of linkage disequilibrium (LD) indicated there existed a correlation between specific genotypes. We observed linkage disequilibrium in the four loci, among which a strong linkage disequilibrium was found between rs2157598 and rs5761664 (D' = 0.99, *r*^2^ = 0.71) (Fig. 1).

**Table 3:** Haplotype analysis of MIAT polymorphisms and IS risk

rs2157598	rs5761664	rs1894720	rs9625066	Controls (%)	IS (%)	OR (95% CI)	*P* value
A	C	G	A	100 (16.1)	80 (12.6)	0.75 (0.55–1.03)	0.074
A	C	G	C	205 (33.1)	252 (39.4)	1.32 (1.05–1.67)	0.017
A	C	T	C	45 (7.3)	47 (7.3)	1.00 (0.66–1.53)	0.993
T	C	G	A	51 (8.2)	49 (7.7)	0.93 (0.62–1.40)	0.722
T	T	G	C	22 (3.6)	19 (2.9)	0.82 (0.44–1.53)	0.531
T	T	T	C	193 (31.2)	187 (29.3)	0.92 (0.72–1.17)	0.495

*IS* Ischemic stroke, *OR* Odds ratio, *95% CI* 95% confidence interval

Only frequencies greater than 1% are listed

### Analysis of risk factors for IS

Logistic regression was applied to analyze the risk factors for IS, and the results are listed in Table 4. The risk factors included: TG (OR = 1.49, 95%CI, 1.26-1.77), TC (OR = 1.65, 95%CI, 1.28–2.11), HDL-C (OR = 0.02; 95%CI, 0.01-0.04), LDL-C (OR = 0.98, 95% CI, 0.76-1.25) and rs9625066AA/AC (OR = 0.55; 95% CI, 0.37-0.83) (*P* < 0.05). Logistic regression analysis exhibited that the TC, TG and HDL-C were still associated with the risk of IS.

**Table 4:** Logistic regression analysis of risk factors

Variables	B	S. E	*P* value	OR (95%CI)
TG	0.339	0.086	< 0.001	1.49 (1.26-1.77)
TC	0.498	0.128	< 0.001	1.65 (1.28-2.11)
HDL-C	-3.902	0.380	< 0.001	0.02 (0.01-0.04)
LDL-C	-0.025	0.126	0.840	0.98 (0.76-1.25)
rs9625066AA/AC	-0.591	0.204	0.004	0.55 (0.37-0.83)

*TG* triglycerides, *TC* total cholesterol, *HDL-C* high-density lipoprotein cholesterol, *LDL-C* low-density lipoprotein cholesterol

### Correlation between MIAT gene SNPs and tissue-specific expression

**Fig. 2:**
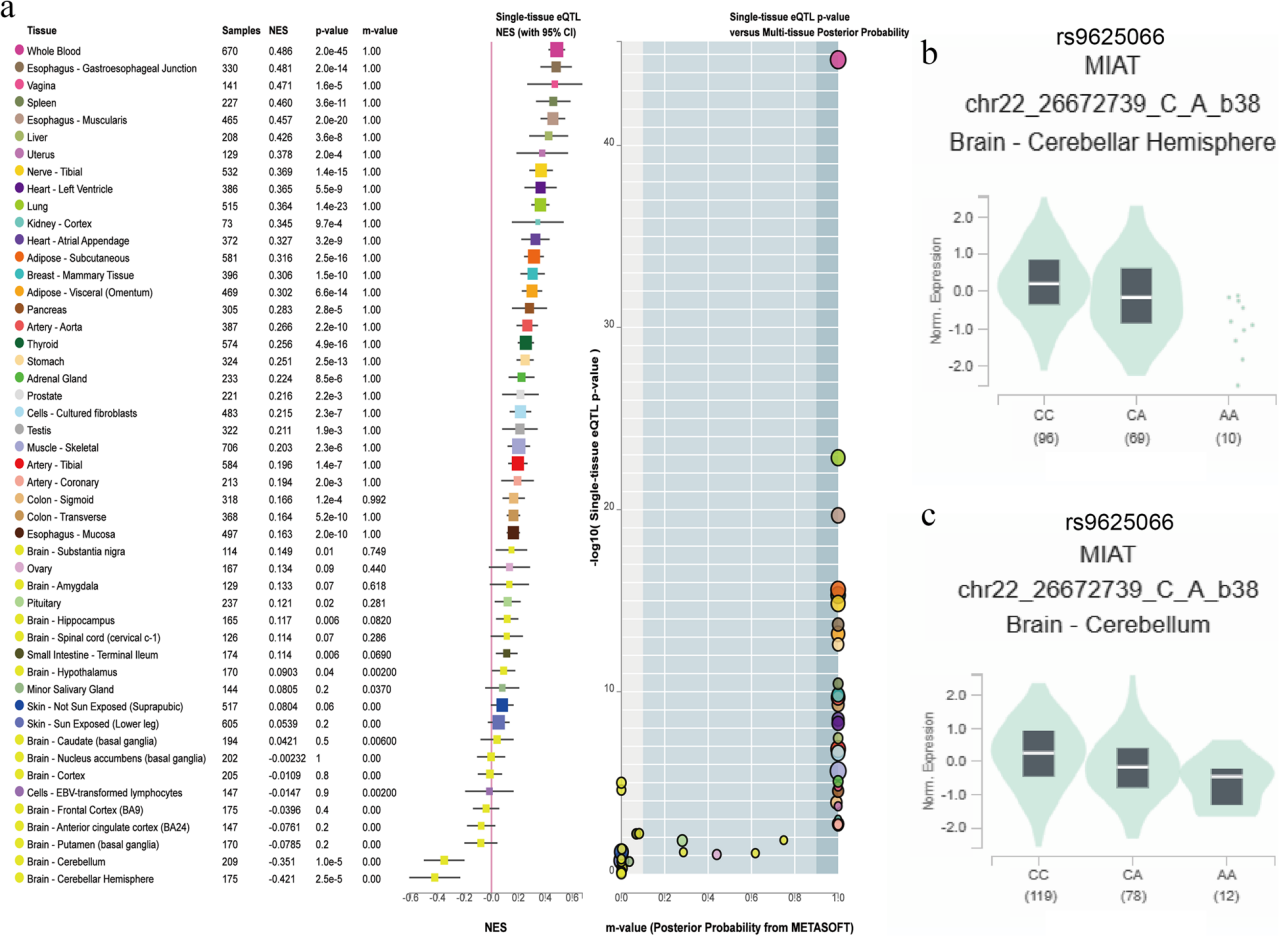
Quantitative trait locus analysis of rs9625066 expression rs9625066 expression in single tissue (**a**), brain-hippocampus (**b**) and brain-cerebellar hemisphere (**c**)

We used GTEx data (https://www.gtexportal.org/home/) to determine the correlation between MIAT gene SNPs and tissue-specific expressions. Expression quantitative trait loci (eQTL) analysis, or expression quantitative trait loci analysis, revealed how genetic variation at a locus can impact the expression levels of specific genes across different tissues. The eQTL analysis showed that the rs9625066 polymorphism was correlated with MIAT expression in tissues, and CC carriers of rs9625066 had elevated MIAT expression in brain tissues, such as cerebral-hippocampus, and cerebral-cerebellar hemispheres (*P* < 0.001) (Fig. 2).

## Discussion

In this study, we examined the association between four single nucleotide polymorphisms (rs2157598, rs5761664, rs1894720, and rs9625066) in the MIAT gene and the risk for the development of IS. We found that individuals carrying the MIAT gene rs9625066 AA/CA genotype and A allele had significantly reduced risk for IS development. However, we failed to found any significant association between rs2157598, rs5761664, rs1894720 and risk for IS. Haplotype analysis revealed that the (A-C-G-A) haplotype increased the risk of IS development by 1.32-fold. Logistic regression analysis identified some independent impact factors for IS including rs9625066 AA/AC, TC, TG, HDL-C. These findings suggest that MIAT rs9625066 might etiologically contributed to the pathogenesis of IS.

Initially identified as a lncRNA in 2006, MIAT is a polyadenylated transcript, measuring roughly 10 kb and containing seven exons [16, 17]. MIAT is highly conserved in mammals, where it accumulates in the nucleus and is expressed in the central nervous system, heart, lungs and spleen [18]. The role of MIAT in neurological diseases is reportedly regulated by miRNAs. Specifically, upregulated miR-204-5p could effectively mitigate the injury of cerebral microvascular endothelial cells by silencing MIAT expression, while inducing neovascularization and significantly increasing the number of surviving neurons [19]. Overexpression of MIAT exacerbated the impaired behavioral activity and neurological function in mice by competitively binding to miR-874-3p, causing neuronal apoptosis and up-regulating the expression of inflammatory factors [20]. In addition, MIAT promoted the proliferation of human carotid smooth muscle cells through the ERK-ELK1-EGR1 pathway, thereby playing a pivotal role in the development and destabilization of atherosclerotic plaques [21]. It has also been found that MIAT expression was significantly elevated in atherosclerotic plaques, and this plaque buildup led to lumen narrowing, which, in turn, contributed to the development of IS [22]. A study by Zhu et al. also confirmed that MIAT was significantly upregulated in IS patients, further supporting that MIAT might be involved in the development and progression of IS [23]. Overall, MIAT might act as an important regulator in a variety of neurological diseases, especially in the development of IS.

In recent years, the association between lncRNA-related polymorphisms and disease risk has become a topic of active investigations. Since IncRNA is functionally-diverse, MIAT-associated SNPs have attracted the attention of researchers [24, 25]. For example, Ma et al. explored the relationship between nine MIAT SNPs and acute myocardial infarction (AMI) in the Chinese Han population and confirmed that patients carrying the rs5752375 TT genotype had a 3.91-fold increased risk of developing AMI compared to the carriers of CC genotype [26], and rs1894720 of MIAT was significantly associated with paranoid schizophrenia, with AA carriers exhibiting an increased risk for this disorder [27]. Furthermore, Li and his colleagues and others found that subjects with the GT and TT genotypes of rs1894720 were at a higher risk of age-related cataracts [28]. These studies implied that functional SNPs in MIAT could serve as potential indicators of related diseases. Globally, IS has become a major public health concern due to the unavailability of early diagnostic markers, and a high mortality rate. In recent years, with the development of molecular biology and the completion of the Human Genome Project, some advances have been made in the regulation of the expression of SNPs of lncRNAs in diseases [29–31]. For example, the rs145204276 del allele of lncRNA- GAS5 was found to raise the risk of IS by increasing the transcriptional activity and the expression of rs145204276 of lncRNA-GAS [32]. Rezaei et al. found that the rs217727 recessive model of lncRNA H19 increased the risk of IS by 2.80-fold [33]. Moreover, the lncRNA-MALAT1 rs619586 AA and rs3200401 CT, TT genotypes were associated with an elevated risk of IS, whereas the lncRNA-ANRIL rs10965215 GG genotype was found to be protective against IS [34]. These studies provided new insights into the genetic mechanisms of IS. We further confirmed the hypothesis that SNPs of MIAT are associated with the risk of IS, and found that individuals with rs9625066 AA/CA genotypes and the A allele significantly lowered the risk for the development of IS. The A-C-G-A haplotype was associated with a reduced risk of IS development. Logistic regression also showed that the rs9625066 AA/CA exerted an effect on risk for IS. Additionally, the GTEx database search exhibited that subjects with the rs9625066 CC genotype had higher levels of MIAT expression in brain-hippocampus and brain-cerebellar hemisphere. The exact mechanisms underlying the findings need to be confirmed by further studies. In conclusion, the rs9625066 in MIAT might serve as a new target for the treatment of IS.

This study revealed a correlation between MIAT gene polymorphisms and IS. However, some limitations of this study have to be acknowledged. Firstly, we included samples from hospitals in the same region, which rendered it difficult to completely eliminate the possibility of selection bias. Secondly, due to the limited clinical data of the patients, we were unable to obtain relevant information on smoking and alcohol consumption, which prevented the further analysis of gene-environment interactions. In addition, the relatively small sample size might affect the power of our findings. Therefore, a larger sample size is needed to further validate the role of MIAT SNPs in IS susceptibility.

## Conclusions

In conclusion, we demonstrated a significant association between lncRNA-MIAT rs9625066 and IS in the Chinese population. The rs9625066 A allele contributed to a reduced risk of IS, whereas the C allele increased the risk of developing IS. These findings suggest that rs9625066 may be a potential biomarker for the occurrence and development of IS.

## Data Availability

All data generated or analyzed during this study are included in this published article.
